# Translating non-coding genetic associations into a better understanding of immune-mediated disease

**DOI:** 10.1242/dmm.049790

**Published:** 2023-03-07

**Authors:** Christina T. Stankey, James C. Lee

**Affiliations:** ^1^ Genetic Mechanisms of Disease Laboratory, The Francis Crick Institute, London NW1 1AT, UK; ^2^ Department of Immunology and Inflammation, Imperial College London, London W12 0NN, UK; ^3^ Institute of Liver and Digestive Health, Royal Free Hospital, University College London, London NW3 2PF, UK

**Keywords:** Genetics, Genomics, Immune-mediated disease, Immunology

## Abstract

Genome-wide association studies have identified hundreds of genetic loci that are associated with immune-mediated diseases. Most disease-associated variants are non-coding, and a large proportion of these variants lie within enhancers. As a result, there is a pressing need to understand how common genetic variation might affect enhancer function and thereby contribute to immune-mediated (and other) diseases. In this Review, we first describe statistical and experimental methods to identify causal genetic variants that modulate gene expression, including statistical fine-mapping and massively parallel reporter assays. We then discuss approaches to characterise the mechanisms by which these variants modulate immune function, such as clustered regularly interspaced short palindromic repeats (CRISPR)-based screens. We highlight examples of studies that, by elucidating the effects of disease variants within enhancers, have provided important insights into immune function and uncovered key pathways of disease.

## Teaser

**Summary:** The success of genetic studies provides a unique opportunity to better understand immune-mediated disease mechanisms. To achieve this, however, knowledge of where disease-associated variants lie must be translated into an understanding of how they cause disease.

## Introduction

Autoimmune and inflammatory diseases are a heterogeneous set of complex disorders characterised by an aberrant immune response that damages host tissue. These immune-mediated diseases – which range from inflammatory bowel disease to multiple sclerosis to systemic lupus erythematosus – affect up to 10% of individuals in Western countries and are rapidly increasing in incidence in other parts of the world (Freeman et al., 2021; King et al., 2020; Patterson et al., 2019; Safiri et al., 2019; Walton et al., 2020). Almost all of these conditions require better treatments, but the attrition rate of drugs entering clinical development is high – mainly because our understanding of disease aetiology and mechanisms remains incomplete.

Familial patterns of inheritance have long suggested a genetic contribution to immune-mediated diseases. Indeed, despite a growing appreciation of the environmental factors involved in disease development, genetics remains the strongest risk factor for most immune-mediated conditions. Early linkage studies identified a handful of genetic associations, typically with large effect sizes. For example, several studies detected hits at the human leukocyte antigen (HLA) locus, such as the association of *HLA-DQA1* and *HLA-DQB1* haplotypes (see Glossary, Box 1) with coeliac disease and of *HLA-DQB* with type 1 diabetes mellitus (Sollid et al., 1989; Todd et al., 1987). A smaller number of studies also identified novel susceptibility genes, such as nucleotide-binding oligomerisation domain-containing protein 2 (*NOD2*) in Crohn's disease (Hugot et al., 2001). However, the genetic basis of most immune-mediated diseases remained largely unknown until the advent of genome-wide association studies (GWAS; Box 1). This technological advance made it possible to detect disease-associated genetic variants with modest effect sizes that collectively account for a large proportion of disease risk (Visscher et al., 2017). Fuelled by large sample sizes and international collaborations, GWAS rapidly identified hundreds of loci associated with immune-mediated diseases [Anderson et al., 2011; International Genetics of Ankylosing Spondylitis Consortium (IGAS) et al., 2013; Tsoi et al., 2012]. Collectively, these results demonstrated the following: (1) the polygenic nature of most immune-mediated diseases, where many common genetic variants individually contribute a small amount to disease susceptibility; (2) the widespread role of pleiotropy (Box 1), where a genetic haplotype confers susceptibility to multiple immune-mediated diseases; and (3) the importance of core pathways, where immune-mediated disease associations cluster within key biological processes such as T-cell activation or cytokine signalling (Cotsapas et al., 2011; Ellinghaus et al., 2016; Schmiedel et al., 2018). In a few cases, GWAS identified susceptibility variants in pathways not previously thought to be involved in a particular disease and thereby generated new insights into pathobiology. This is best exemplified by the characterisation of defective autophagy in Crohn's disease pathogenesis (Hampe et al., 2007; Rioux et al., 2007). Despite this success, however, the molecular basis of most genetic associations – either with immune-mediated diseases or complex diseases more generally – remains unresolved. Indeed, translating the success of GWAS into an improved understanding of disease mechanisms is arguably the greatest challenge in modern human genetics.

**Box 1. Glossary**

**4C:** a high-resolution ‘one-versus-all’ approach to map chromatin interactions. Following cross-linking, digestion, and ligation, chimeric DNA fragments are circularised and PCR amplified using primers specific to one locus of interest. High-throughput sequencing is then used to identify all fragments interacting with this locus.

**Allelic imbalance:** a phenomenon in a heterozygous individual where some measure of the two alleles diverges from the expected 1:1 ratio for a particular cellular trait.

**Assay for transposase-accessible chromatin sequencing (ATAC-seq):** a method to assess chromatin accessibility that uses a hyperactive Tn5 to simultaneously cleave open chromatin and insert adaptors for high-throughput sequencing. Sequenced reads are mapped to identify regions of open chromatin.

**Bayesian methods:** a set of statistical methods that estimate the probability of a hypothesis based on existing data and the prior probability of the hypothesis. In statistical fine-mapping, Bayesian methods are used to calculate the posterior probability that a variant is causal given the linkage disequilibrium structure of the locus.

**Capture-C:** a high-resolution ‘many-versus-all’ approach to map chromatin interactions. Following cross-linking, digestion and ligation, loci of interest are enriched through pulldown with oligonucleotide probes. High-throughput sequencing is then used to identify fragments interacting with these loci.

**Chromatin interaction analysis with paired-end tag (ChIA-PET):** a high-resolution method to identify interacting chromatin bound by a protein of interest. Chromatin is cross-linked, fragmented and immunoprecipitated to obtain DNA–protein of interest complexes. DNA is ligated with biotinylated adaptors, then is pulled down and sequenced to identify chromatin interactions with specific proteins.

**Credible set:** the smallest set of variants that contains the true causal variant(s) at a defined probability.

**DNase I hypersensitivity sites sequencing (DNase-seq):** a method to assess chromatin accessibility whereby nuclei are digested with DNase I, an enzyme that cleaves chromatin at regions free of nucleosomes or transcription factors. Cleaved fragments are sequenced and mapped to identify DNase hypersensitive sites, which denote regions of open chromatin.

**Genome-wide association studies (GWAS):** an experimental approach to identify associations between genotype and phenotype. Study populations are selected and genotyped using microarrays or whole-genome sequencing, then the genotyped variants are tested for association with the phenotype of interest.

**Haplotype:** a combination of alleles at a locus that tend to be inherited together.

**Hi-C:** a lower-resolution ‘all-versus-all’ approach to map chromatin interactions. Following cross-linking and digestion, biotin-tagged ligation fragments are generated. Biotinylated fragments are enriched by pulldown, and high-throughput sequencing of the fragments is used to identify all interacting pairs of loci.

**Insulator:** a regulatory element that functions either by blocking interactions of enhancers and promoters or by preventing spread of heterochromatin.

**Jurkat-dCas9-VP64 cells:** an immortalised human CD4^+^ T-cell line that constitutively expresses dCas9-VP64.

**Mediator:** a key transcriptional co-activator complex at enhancers.

**Nuclear factor kappa B (NF-κB):** a key transcriptional mediator of inflammatory processes across immune lineages.

**Pleiotropy:** the association of a single genotype with multiple phenotypes.

**Protospacer adjacent motif (PAM):** a short nucleotide sequence that is necessary for recognition and cleavage of the target sequence by a Cas protein. The PAM sequence depends on the Cas protein used.

**Quantitative trait loci (QTL):** loci at which a genetic variant is associated with a measurable phenotypic trait.

**Regulatory T (Treg) cells:** a subpopulation of CD4^+^ T cells that suppresses inflammatory processes and contributes to maintenance of immunological tolerance.

**RNA editing:** a process that prevents formation of immunogenic double-stranded RNA molecules.

**RNA splicing:** a process that generates different transcript isoforms and can affect transcript stability.

**Tiling:** an approach for designing constructs for a high-throughput screen to cover a genomic region of interest at even intervals. Constructs are usually designed with an offset of one or a few base pairs to ensure high-resolution characterisation of the region.

Most immune disease-associated genetic variants map to non-coding regions of the genome and are particularly enriched in immune cell enhancers – regulatory sequences that interact with promoters and transcriptional machinery to increase the expression of target genes (Ernst et al., 2011; Farh et al., 2015; Maurano et al., 2012). Disease-associated variants within enhancers have therefore been hypothesised to disrupt transcriptional circuits and dysregulate key processes in immune cell biology (Javierre et al., 2016). Identifying the molecular and cellular mechanisms involved could provide important insights into immune regulation and disease biology. However, attempts to mechanistically characterise regulatory variants have been hindered by the need to identify the target gene(s), the cell types in which these gene(s) are dysregulated and the conditions under which this dysregulation occurs.

Recently developed genetic and genomic techniques have now made it possible to elucidate the effects of genetic variants within enhancers and thus provide a means to translate GWAS associations into an understanding of disease biology (Bourges et al., 2020; Nasrallah et al., 2020; Simeonov et al., 2017). Here, we review these techniques in the context of immune-mediated disease. We describe approaches to identify putative causal variants, including statistical methods, such as fine-mapping and colocalisation with chromatin features, and experimental methods, such as high-throughput reporter assays. We also review approaches to resolve the biological effects of regulatory variants in immune-mediated diseases, including chromatin interaction maps and clustered regularly interspaced sport palindromic repeats (CRISPR)-based screens. Furthermore, we provide examples of recent studies that have mechanistically linked genetic variation in enhancers to immune dysregulation, highlighting the potential for genetics to reveal key pathways in disease biology and identify new therapeutic targets.

## Challenges to translating associations into disease mechanisms

**Fig. 1.**
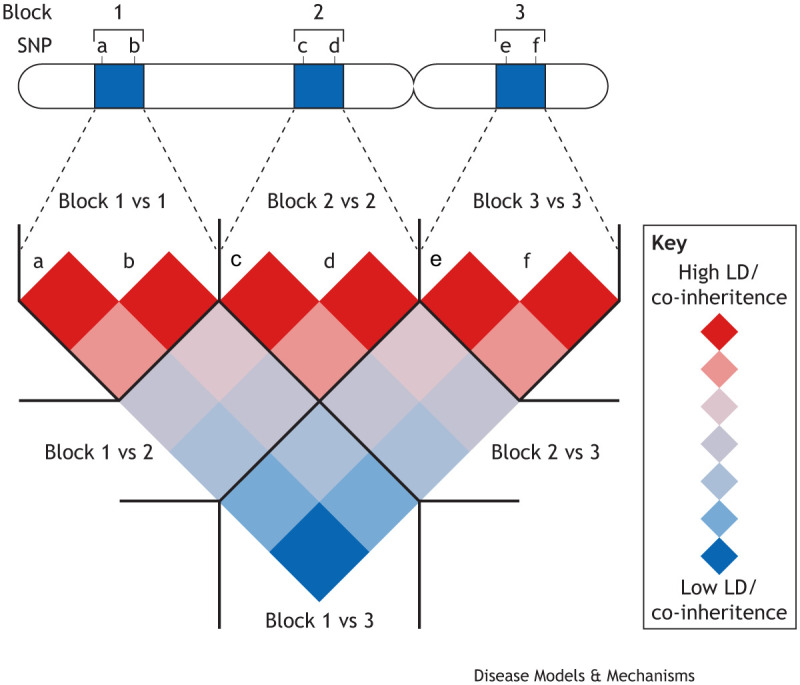
**Haplotype blocks and linkage disequilibrium.** Haplotype blocks (blue rectangles, top; 1, 2 and 3) are regions on a chromosome (top) that are inherited as a unit, such that variants or single-nucleotide polymorphisms (SNPs; a, b, c, d, e and f) within the blocks tend to be inherited together. This gives rise to linkage disequilibrium (LD), where inheritance of common genetic variants within a haplotype block is highly correlated (red diamonds; high LD/co-inheritance, e.g. Block 1 vs 1), whereas inheritance of variants in different haplotype blocks is weakly correlated (light-red/light-blue diamonds; weak LD/co-inheritance, e.g. Block 1 vs 2) or not correlated (blue diamonds; low LD/co-inheritance, e.g. Block 1 vs 3).

Two key challenges must be overcome to successfully translate genetic associations into a better understanding of immune disease mechanisms. First, we need to identify the causal variants for which functional consequences directly alter disease risk. This is not a trivial task because an associated locus can contain tens to hundreds of candidate variants. Ironically, this problem results from the haplotype structure of the human genome, which was what enabled the development of GWAS in the first place (Daly et al., 2001). Specifically, the human genome is structured into ‘haplotype blocks’, genomic regions spanning tens to hundreds of kilobases that are typically inherited as single units during meiosis (Fig. 1) (Gabriel et al., 2002). As such, single-nucleotide polymorphisms (SNPs) within a haplotype block are frequently inherited together, a phenomenon that is known as ‘linkage disequilibrium’ (LD) (Hästbacka et al., 1992). The allelic variation at any one SNP within a haplotype block can therefore be used to infer the variation at all other SNPs in the block. This observation paved the way for GWAS because it meant that genome-wide genetic variation could be captured by genotyping only a subset of all SNPs (Altshuler et al., 2008). However, it also means that, for any disease-associated locus, there are often many candidate SNPs that could be responsible for the association.

Second, we need to characterise the functional consequences of causal variants on downstream biological pathways. This challenge is not straightforward because only a small proportion of disease-associated variants map to protein-coding regions of the genome (Gusev et al., 2014; Hindorff et al., 2009). Indeed, although we now know that most non-coding disease variants fall within putative enhancers, the specific genes that these enhancers regulate are often unclear, with some variants lying near multiple plausible candidate genes and others residing within intergenic regions that contain no genes (Mifsud et al., 2015; Onengut-Gumuscu et al., 2015). Even when a target gene can be identified, many enhancers exhibit both cell-type- and cell-state-specific activity (Farh et al., 2015). Accordingly, both the appropriate cell type and the disease-relevant external stimuli/conditions must be determined before the functional consequences of a causal variant can be fully resolved.

## Challenge 1: identifying the causal variant

A variety of approaches have been developed to identify causal variants at disease-associated loci. These include statistical methods to nominate putative causal variants based on patterns of association, methods to compare the location of candidate variants with known functional genomic regions, and experimental methods to directly assess variants for gene expression-modulating activity. The advantages and limitations of these approaches are summarised in Table 1.

**Table 1.**
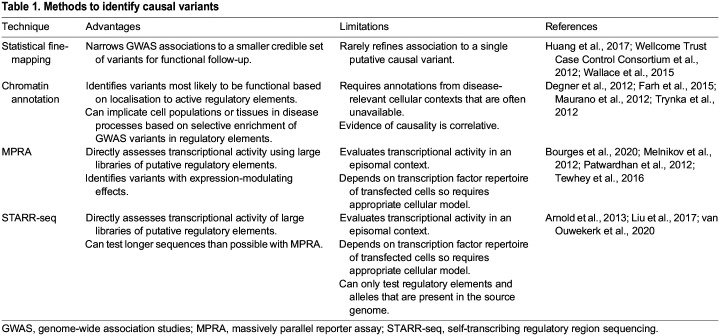
Methods to identify causal variants

### Statistical fine-mapping

**Fig. 2.**
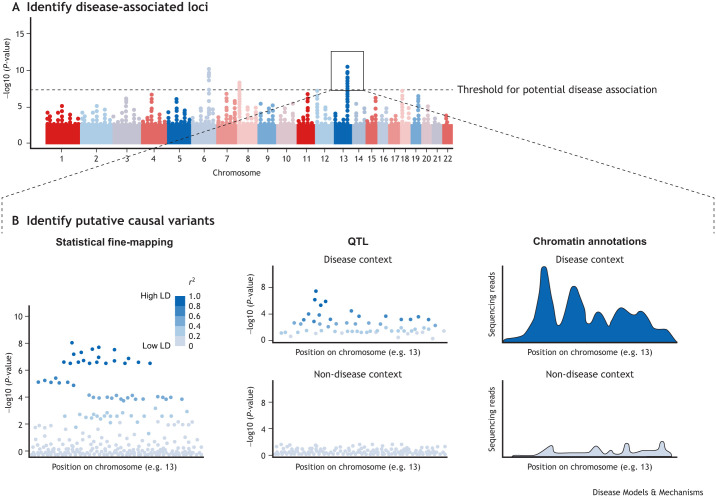
**Statistical methods to identify putative causal variants.** (A) First, genome wide-association studies (GWAS) identify loci associated with a disease. Single-nucleotide polymorphisms (SNPs) within these loci that reach a corrected *P*-value threshold (dashed line) are associated with the disease. An association signal (for example on chromosome 13) can be refined through various methods to identify putative causal SNPs, as shown in B. (B) Statistical fine-mapping (left) can be used to refine disease associations. This technique typically uses Bayesian methods and data from SNP microarrays and whole-genome sequencing to evaluate the probability that each variant is causal given the haplotype structure across the locus. SNPs (dots) are coloured according to the *r*^2^ value, which denotes linkage disequilibrium (LD) with the lead SNP at the locus (*r*^2^ values closer to 1 indicate a higher LD). Corrected *P*-values are adjusted for multiple testing. Quantitative trait loci (QTL; middle) are loci in which genetic variation is associated with a cellular trait. In the plots, the corrected *P*-value indicates the association of SNPs with the trait of interest, with higher associations in disease than in non-disease contexts. Chromatin annotations (right) denote, for example, chromatin accessibility or histone marks that are indicative of enhancer activity. In disease context, higher corrected *P*-values indicate higher chromatin accessibility.

The most common approach used to identify causal variants at disease-associated loci is statistical fine-mapping (Fig. 2). This technique seeks to refine a genetic association to a smaller subset of credible variants, typically by using Bayesian methods (Box 1) to evaluate the probability that each variant is causal given the haplotype structure across the locus (Wellcome Trust Case Control Consortium et al., 2012; Wallace et al., 2015). Because association statistics for each SNP at the locus must be known, dense genotyping data are required for this analysis. Statistical fine-mapping efforts have therefore benefitted from the development of Immunochip, an SNP microarray that provides dense genotyping of almost 200 human loci associated with at least one autoimmune or inflammatory disease (Cortes and Brown, 2011). Statistical fine-mapping has also benefitted from improvements in imputation reference panels, a set of reference haplotypes generated by whole-genome sequencing (WGS) that are used to predict genotypes of variants not included on the SNP microarray of a GWAS (Kowalski et al., 2019; McCarthy et al., 2016). Use of reference panels – or, as is becoming increasingly common, direct WGS of case-control samples – provides comprehensive genotype data and thereby also enables detection of rare variants (Almöf et al., 2019; Taliun et al., 2021). With these advances, statistical fine-mapping has helped resolve a small number of GWAS loci down to a single variant in diseases such as ankylosing spondylitis, rheumatoid arthritis and ulcerative colitis [International Multiple Sclerosis Genetics Consortium (IMSGC) et al., 2013; International Genetics of Ankylosing Spondylitis Consortium (IGAS) et al., 2013; Eyre et al., 2012; Trynka et al., 2011; Tsoi et al., 2012]. Importantly, however, strong LD (i.e. high rates of co-inheritance) between candidate SNPs has meant that this approach is not possible for most loci [International Multiple Sclerosis Genetics Consortium (IMSGC) et al., 2013; de Lange et al., 2017; Eyre et al., 2012; Huang et al., 2017]. As such, alternative approaches are needed to identify causal variants.

One way to increase the resolution of statistical fine-mapping is to draw on GWAS data from ethnically diverse populations. This approach, referred to as trans-ethnic meta-analysis, was first adopted to compare the genetic architecture of complex diseases across populations and to improve statistical power for locus discovery (Keller et al., 2014; Kim et al., 2015; Liu et al., 2015; Okada et al., 2014; Wang et al., 2010; Yin et al., 2015). Methods for trans-ethnic fine-mapping were later developed to leverage differences in the haplotype structure across different populations to help pinpoint putative causal variants (Kichaev and Pasaniuc, 2015; Mägi et al., 2017; Morris, 2011). These methods exploit the fact that LD structure is determined by ancestral meiotic recombination events and so varies among human populations. At loci at which a disease association is shared but the genetic architecture differs among ancestrally diverse populations, trans-ethnic fine-mapping can decipher the disease association signal by narrowing down the number of candidate variants to only those that are shared in both populations. Although few such studies have been performed, which reflects the fact that most GWAS have been conducted in European populations, these have successfully narrowed credible sets (Box 1) of putative causal variants for several complex traits and diseases, including several immune-mediated diseases (Chen et al., 2020; Kichaev and Pasaniuc, 2015; Liu et al., 2016; Ota et al., 2021; Stuart et al., 2022; van Rooij et al., 2017; Wojcik et al., 2019). For instance, in a GWAS meta-analysis of rheumatoid arthritis, trans-ethnic fine-mapping reduced the average size of credible sets by 20% (Kichaev and Pasaniuc, 2015). This highlights the benefits and importance of recruiting diverse populations for studies of genetic disease risk.

### Colocalising candidate SNPs with complementary datasets

Another way to reduce the number of candidate variants at a disease-associated locus is to integrate the association statistics with other relevant genetic data. Aside from facilitating the discovery of disease susceptibility loci, the advent of affordable genome-wide genotyping has made it possible to study the consequences of genetic variation on a range of cellular traits. These include genetic effects on levels of mRNA, proteins, histone modifications, chromatin accessibility and DNA methylation – referred to as quantitative trait loci (QTL; Box 1). Because most GWAS signals are non-coding and likely to act by perturbing gene regulation, data from QTL studies, which frequently relate to, or reflect, altered enhancer activity, can help refine credible sets of putative causal variants and investigate their functional consequences.

One common approach is to use colocalisation analysis to investigate the overlap between GWAS associations and QTL data (Fig. 2). Colocalisation methods formally test the hypothesis that individual signals, such as a disease association and an effect on gene expression, co-occur at a given locus because of a shared causal variant(s) – a finding that would implicate both the variant and the biological effect in disease pathology (Giambartolomei et al., 2014; Hormozdiari et al., 2016; Wen et al., 2017). This approach has been successfully used to nominate candidate genes for dozens of GWAS loci in immune-mediated diseases, but has been less successful in identifying causal variants because both the GWAS and QTL datasets remain constrained by LD between candidate SNPs (de Lange et al., 2017; Guo et al., 2015; Okada et al., 2014; Peters et al., 2016; Wallace et al., 2012). A further challenge is that in immune cells most of the cellular features measured by QTL studies are highly dependent on both cell lineage and activation state (Chun et al., 2017; Fairfax et al., 2014; Kim-Hellmuth et al., 2017; Yazar et al., 2022). Accordingly, GWAS–QTL colocalisation studies need to be performed in the relevant cell type under suitable stimulation conditions to avoid missing evidence of a shared causal variant. This is an important limitation, as most QTL datasets have been generated from whole-blood samples or from immortalised cell lines that poorly reflect the physiology of primary immune cells (Andreu et al., 2017; Bartelt et al., 2009). Moreover, even when studies in specific immune cell types have been conducted, most have used unstimulated cells, which often differ in physiology from that of cells exposed to disease-relevant stimuli (Kerimov et al., 2021).

### Combining GWAS data with chromatin features

A complementary set of methods have used known genomic features, often based on chromatin annotations, to try to interpret GWAS signals (Fig. 2) (Maurano et al., 2012; Pickrell, 2014). Because transcriptional regulation often relies on the binding of transcription factors, co-activators and other transcriptional machinery, chromatin that is inaccessible to these molecules (i.e. heterochromatinised) is unlikely to harbour SNPs that causally affect gene regulation. An important caveat, however, is that this assumes that the correct cell type is being studied and that the causal variant affects transcription factor binding, rather than other aspects of transcriptional control, such as insulator (Box 1) function. Nevertheless, high-throughput methods to annotate chromatin states have emerged as a useful tool to refine lists of candidate SNPs. Two general approaches have been adopted. One approach is to identify chromatin that is accessible to transcription factor binding using sequencing-based methods, such as DNase I hypersensitivity sites sequencing (DNase-seq; Box 1) or assay for transposase-accessible chromatin sequencing (ATAC-seq; Box 1) (Boyle et al., 2008; Buenrostro et al., 2015; Song et al., 2011; Thurman et al., 2012; Tong et al., 2016). A second approach is to use histone modifications that are associated with specific regulatory features, such as histone H3 lysine 27 acetylation (H3K27ac) or histone H3 lysine 4 monomethylation (H3K4me1), to identify enhancers (Creyghton et al., 2010; Heintzman et al., 2009). Both of these approaches have shown that the chromatin landscape is also cell-type specific, prompting the development of models to help prioritise pathogenic cell types based on selective enrichment of disease-associated variants in active regulatory regions (Ernst et al., 2011; Song et al., 2011; Thurman et al., 2012). This in turn led to the realisation that T-cell subsets frequently show the strongest enrichment for immune disease-associated variants (Farh et al., 2015; Maurano et al., 2012; Onengut-Gumuscu et al., 2015; Pickrell, 2014; Schmidt et al., 2015; Trynka et al., 2011). As with QTLs, however, the enrichment of immune disease-associated variants is most pronounced – and in some cases, only detectable – in certain primary cell types under specific stimulation conditions (Calderon et al., 2019; Glinos et al., 2020; Soskic et al., 2019). This finding again reflects the context-specific function of many enhancers and highlights the need for GWAS associations to be interpreted in disease-relevant cells under appropriate conditions, many of which remain incompletely catalogued at present.

### Testing candidate SNPs for expression-modulating effects

**Fig. 3.**
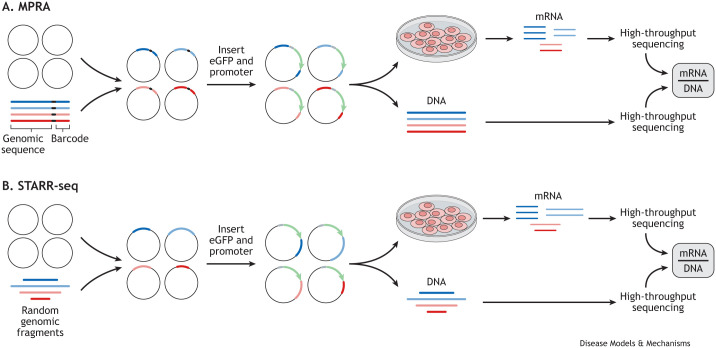
**Experimental methods to identify variants that modulate gene expression.** (A,B) Massively parallel reporter assay (MPRA; A) and self-transcribing active regulatory region sequencing (STARR-seq; B) are used to directly assess the transcriptional effects of putative regulatory elements and/or putative causal variants. A library of barcoded putative enhancer sequences (MPRA) or genomic fragments (STARR-seq) are cloned into empty vectors (circles). A promoter (grey) and enhanced green fluorescent protein (eGFP; green arrow) are also inserted. The resulting plasmid is transfected into cells, RNA is extracted, and mRNA barcode counts (MPRA) or mRNA genomic fragment counts (STARR-seq) are obtained by high-throughput sequencing. mRNA counts are then normalised to their corresponding DNA counts within the transfected plasmid pool. This provides a measure of expression-modulating activity (mRNA/DNA).

An important caveat to the methods described above, which seek to refine lists of candidate SNPs using external datasets, is that the results produced are correlative and do not show whether a putative causal variant actually modulates gene expression. To address this issue, individual genetic reporter assays have been used to directly assess the transcriptional effects of putative causal SNPs (Elsby et al., 2010). However, these assays were traditionally labour intensive and low throughput, often assessing one allele at a time, and were therefore unsuitable for evaluating the possible functional consequences of thousands of candidate variants across many disease-associated loci. More recently, high-throughput assays of enhancer activity have been developed that can simultaneously test the regulatory effects of multiple non-coding sequences. Two main approaches have been used (Fig. 3). The first method, massively parallel reporter assay (MPRA), involves synthesising a library of short DNA oligonucleotides that can be systematically tested for enhancer/repressor activity (Melnikov et al., 2012; Patwardhan et al., 2012). This is achieved by cloning the DNA oligonucleotides into a reporter plasmid such that the putative enhancer sequence lies immediately upstream of a promoter and a reporter gene, similar to a luciferase assay. The key difference is that each sequence is paired with a unique oligonucleotide barcode positioned within the 3′ untranslated region (UTR) of the reporter gene, to allow the RNA molecules transcribed from individual plasmids to be matched to the putative enhancer sequence that modulated their expression using barcode sequencing (Fig. 3A) (Kheradpour et al., 2013; Tewhey et al., 2016). In this way, large numbers of putative enhancer sequences can be simultaneously assessed for transcriptional activity by normalising the RNA barcode counts that are obtained from cells following transfection to the corresponding DNA barcode counts within the transfected plasmid pool (Melnikov et al., 2014). The second method, self-transcribing regulatory region sequencing (STARR-seq), adopts a similar approach, but obtains putative enhancer sequences by randomly shearing genomic DNA to create smaller fragments, rather than by synthesising oligonucleotides (Arnold et al., 2013). These genomic fragments are ligated with adaptors and amplified with PCR before being cloned into reporter vectors and transfected into cells. The fragments are then sequenced directly rather than using oligonucleotide barcodes (Fig. 3B). STARR-seq allows longer DNA sequences to be tested than is possible with MPRA, owing to technical limitations with oligonucleotide synthesis, but is constrained by the genotype of the source genome and can only test for expression-modulating effects if both alleles are present.

Both MPRA and STARR-seq have the advantage of directly measuring the process by which non-coding variants principally act, and have been successfully used to distinguish expression-modulating variants from co-inherited non-functional variants (Bourges et al., 2020; Choi et al., 2020; Liu et al., 2017; Lu et al., 2021; Tewhey et al., 2016; Ulirsch et al., 2016; van Ouwekerk et al., 2020). However, although these assays represent powerful tools for characterising the functional effects of disease-associated variants, they too have limitations (Table 1). For example, both MPRA and STARR-seq assess the transcriptional activity of candidate enhancer sequences in an episomal vector using a standard promoter, and so do not fully recapitulate the interactions that take place between specific enhancers and their target promoters within the human genome. This might not matter, however, because the observed transcriptional effects do not appear to depend on the promoter used (Ferreira et al., 2016) – an observation consistent with the recent finding that most enhancers activate all promoters by similar amounts (Bergman et al., 2022). Another important consideration is that any expression-modulating activity is determined by the interaction between the tested DNA sequences and the transcription factors present within the transfected cells. For this reason, the cellular context in which disease-associated variants are tested is a key experimental parameter. To date, most MPRA and STARR-seq studies have been performed using immortalised cell lines, which are highly amenable to transfection but differ substantially in physiology and transcriptional regulation from the primary cells that they seek to model (Andreu et al., 2017; Bartelt et al., 2009). To address this, Bourges et al. (2020) developed an adapted MPRA for use in primary human CD4^+^ T cells, having demonstrated that the conventional MPRA vector was unsuitable for use in primary cells. This study confirmed that putative causal variants could be identified via their expression-modulating activity in primary immune cells, even at loci that were unresolvable by statistical fine-mapping, and that the results differed from those obtained in commonly used cell-line models, such as Jurkat T cells. The adapted MPRA system therefore provides a means to identify putative causal variants in disease-relevant cells by directly assaying the process by which they alter disease risk. This approach, however, does not identify the genes affected by any expression-modulating effects, for which complementary methods are required.

Statistical methods to prioritise putative causal variants and experimental methods to determine the transcriptional effects of individual SNPs have thus meant that it is now possible to identify putative causal variants at many disease-associated loci. The challenge then becomes characterising the mechanisms by which these variants drive disease processes.

## Challenge 2: identifying disease mechanisms

Several complementary methods have been developed to characterise the functional consequences of non-coding variants. For immune-mediated diseases, frequently used approaches include descriptive methods to identify putative target genes affected by disease-associated loci, such as maps of three-dimensional chromatin structure and/or colocalisation analysis, as well as experimental methods to establish functional links between enhancers and target genes with CRISPR-based approaches. The advantages and limitations of these approaches are summarised in Table 2.

**Table 2.**
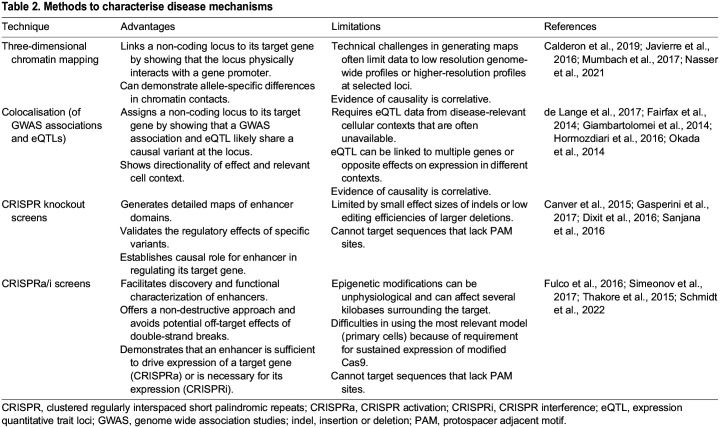
Methods to characterise disease mechanisms

### Combining GWAS data with three-dimensional chromatin interactions

**Fig. 4.**
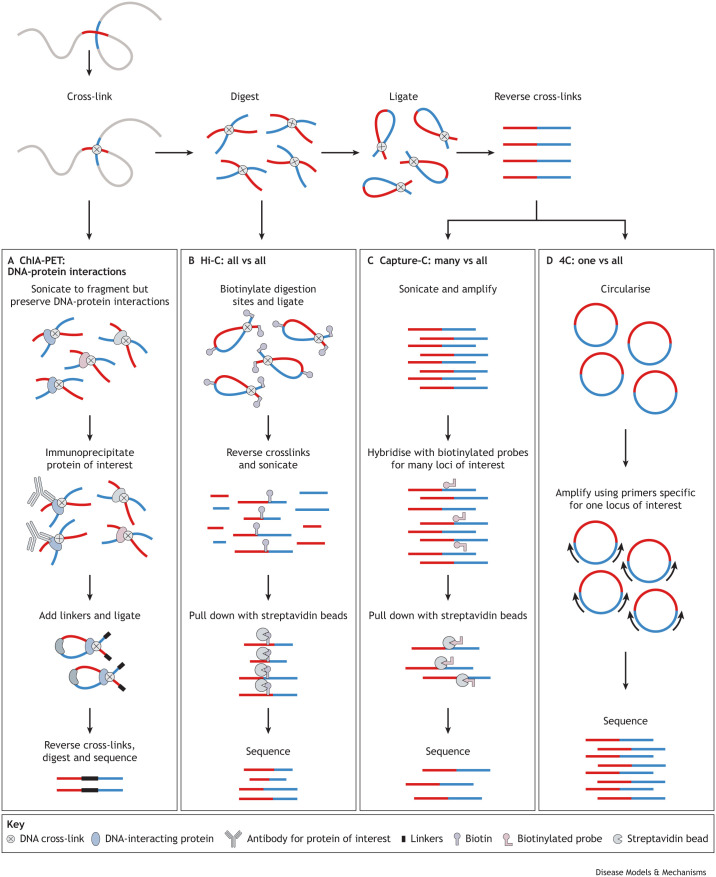
**Methods to map three-dimensional chromatin interactions.** Methods to map three-dimensional chromatin interactions are based on chromatin conformation capture, where chromatin (grey line with red and blue interacting segments) is cross-linked. (A) In chromatin interaction analysis with paired-end tag (ChIA-PET), cross-linked chromatin is sonicated to obtain complexes of DNA fragments and proteins. The protein of interest (blue oval) is immunoprecipitated using the antibody specific for the protein of interest along with the cross-linked DNA. Linkers are added and the fragments are ligated (grey shape). The cross-links are then removed, the proteins are digested, and the DNA is prepared for sequencing to identify fragments of DNA interacting with the protein of interest. (B) In Hi-C, following chromatin digestion to generate cross-linked DNA fragments, biotin is added at the digested ends of DNA fragments, and the fragments are ligated. The cross-links are reversed, then the fragments are sonicated. Streptavidin beads are used to pull down biotinylated fragments. Biotin is removed, and the DNA is prepared for sequencing to identify all interacting pairs of loci. (C,D) In Capture-C and 4C, chromatin is digested to generate cross-linked DNA fragments, which are self-ligated and the cross-links are removed. (C) In Capture-C, ligated fragments are sonicated and amplified by PCR. Biotinylated oligonucleotide probes are added to hybridise to DNA sequences of interest, which are pulled down using streptavidin beads and sequenced to identify fragments interacting with these loci. (D) In 4C, ligated fragments are circularised then are amplified using PCR primers (black arrows) for a locus of interest. High-throughput sequencing is then done to identify all fragments interacting with this locus.

Although early genetic studies typically nominated the closest gene to a disease-associated locus as most likely to be causal, it is now clear that linear distance is not the best predictor of regulatory interactions. Indeed, it is well recognised that chromatin forms three-dimensional loops that can bring enhancers and promoters, which are separated by hundreds of kilobases, into close spatial proximity (Larke et al., 2021; Lieberman-Aiden et al., 2009; Sanyal et al., 2012; Schoenfelder et al., 2015). These interactions can be identified using a series of methods derived from chromatin conformation capture (3C), a technique using which chromatin is cross-linked, enzymatically digested and DNA ligated to produce chimeric fragments containing sequences from loci that were in close spatial proximity (Fig. 4) (Dekker et al., 2002). Common methods include (1) 4C (Box 1), a ‘one-versus-all’ approach to identify all regions interacting with a locus of interest; (2) Capture-C (Box 1), a ‘many-versus-all’ approach to determine the interaction partners for hundreds of loci of interest; (3) Hi-C (Box 1), an ‘all-versus-all’ approach to map all chromatin interactions throughout the genome; and (4) chromatin interaction analysis with paired-end tag (ChIA-PET; Box 1), an approach to assess chromatin interactions anchored by a particular protein (Fig. 4) (Fullwood et al., 2009; Hughes et al., 2014; Li et al., 2012; Lieberman-Aiden et al., 2009; Simonis et al., 2006; Zhao et al., 2006). By enabling unbiased and high-throughput detection of chromatin interactions, these methods have mapped hundreds of non-coding loci to their putative target genes (Hughes et al., 2014; Martin et al., 2015; Meddens et al., 2016; Schoenfelder et al., 2015). Moreover, integrating GWAS data with chromatin interaction maps has shown that disease-associated variants are enriched within interacting enhancers and promoters in disease-relevant cell types (Javierre et al., 2016; Mifsud et al., 2015; Mumbach et al., 2017; Nasser et al., 2021). Allele-specific mapping of disease variants at these loci has identified instances of allelic imbalance (Box 1), where chromatin interactions or accessibility profiles differ for risk and non-risk alleles (Calderon et al., 2019; Mumbach et al., 2017). This allelic imbalance is consistent with the hypothesis that disease variants disrupt transcription factor binding, ultimately leading to altered chromatin state and enhancer activity. Indeed, mechanistic insights from recent chromatin-mapping studies support a model of transcriptional regulation in which master transcription factors facilitate the formation of cell-type-specific regulatory hubs that can encompass many enhancers and promoters and that together drive complex gene expression programs (Di Giammartino et al., 2019; Hsieh et al., 2020; Hua et al., 2021; Oudelaar et al., 2018). Thus, these studies not only implicate genes affected by disease-associated loci but also suggest putative mechanisms of disease, indicating that risk variants within enhancers can perturb the formation of regulatory hubs and reduce or augment transcription of target genes, resulting in dysregulation of cellular processes.

### Identifying causal genes via eQTL data

Another common method used to identify causal genes is to assess the colocalisation of GWAS signals with expression quantitative trait loci (eQTL). eQTL are SNP haplotypes where allelic variants are associated with levels of gene expression (Fairfax et al., 2014). Although such colocalisation analyses cannot always pinpoint causal variants (for reasons described in the earlier section), this approach can provide statistical evidence that a GWAS association and an eQTL are likely to share a mechanistic basis and thus a target gene. An important, limiting, feature of eQTL effects is that they are largely cell-type and cell-state dependent, and so could be missed if the wrong context or cell type is used (Fairfax et al., 2014; Guo et al., 2015; Kim-Hellmuth et al., 2017). Indeed, although GWAS associations are enriched for eQTLs, only a small proportion have been matched to their target gene through colocalisation analysis, likely because eQTL data do not exist for all relevant cell types or conditions. Recently, however, a growing number of datasets have profiled primary human immune cell subpopulations under relevant stimulation conditions, leading to improved detection of colocalisation (Chen et al., 2016; Ota et al., 2021; Schmiedel et al., 2018). This has benefitted from use of single-cell RNA sequencing (scRNA-seq) to identify transcriptional changes that might only occur in very small subsets of cells (Soskic et al., 2022; Yazar et al., 2022). Interestingly, eQTL studies have occasionally uncovered contrasting effects in different cell types, either where different genes are eQTLs for the same GWAS loci or where opposite directional effects are observed (Ota et al., 2021; Peters et al., 2016; Schmiedel et al., 2018). Resolving causal genes in this situation will inevitability require complementary methods.

### Mapping enhancer activity across the genome using CRISPR

In recent years, CRISPR has emerged as a powerful tool for studying genome function, including functionally linking enhancers to target genes. This was initially applied *in vitro* via a conventional knockout approach, in which single-guide RNAs (sgRNAs) were designed to disrupt either non-coding regions around a gene of interest or putative binding sites of a given transcription factor (Canver et al., 2015; Korkmaz et al., 2016; Rajagopal et al., 2016; Sanjana et al., 2016). These studies generated detailed maps of functional enhancers in primary cells and cell lines, facilitating investigation of how specific variants might affect enhancers and establishing mechanistic links between enhancers and their target genes. However, this approach was often limited by the minor effects that small insertions or deletions (indels) have on enhancer function and by the inability to target sequences that lacked protospacer adjacent motif (PAM) (Box 1) sites. To overcome this limitation, subsequent studies used pairs of sgRNAs to create larger deletions, often several hundred bases apart, although this method is, in turn, limited by the lower efficiency of deleting large DNA segments (Diao et al., 2017; Gasperini et al., 2017). Other studies have used a pooled approach to make knockout screens more scalable and better able to detect complex phenotypes. This approach delivers multiple sgRNAs per cell then uses scRNA-seq to determine the effect of each perturbation at a transcript level (Datlinger et al., 2017; Dixit et al., 2016; Jaitin et al., 2016).

An alternative approach was subsequently developed using a nuclease-deactivated Cas9 (dCas9) that was fused to an effector domain that could alter chromatin state at target sites, either activating transcription [CRISPR activation (CRISPRa)] or repressing it [CRISPR interference (CRISPRi)] (Gilbert et al., 2014). This approach has the advantage of being non-destructive and, therefore, allows for assessment of regulatory function while minimising off-target effects due to double-strand breaks. In CRISPRa, dCas9 is fused to a strong transcriptional activator, such as VP64 or VP64-p65-Rta (VPR), making it possible to discover putative enhancers that can drive expression of target genes, including those not typically active under the screening conditions (Dai et al., 2021; Li et al., 2020; Simeonov et al., 2017). In contrast, CRISPRi uses dCas9 fused to a lysine-specific demethylase 1 (LSD1) or Krüppel associated box (KRAB) domain to recruit repressive chromatin modifiers and promote heterochromatin formation (Kearns et al., 2015; Thakore et al., 2015). This approach reduces the activity of enhancers that are functional under the screening conditions and can identify regions necessary for gene expression (Fulco et al., 2016; Klann et al., 2017).

CRISPR-based screens offer a powerful and unbiased approach to identify enhancers in an endogenous context, but also have several limitations (Table 2). First, the epigenetic modifications induced by CRISPRi and CRISPRa are often unphysiological and can alter several kilobases of surrounding chromatin. This makes high-resolution identification of functional enhancer sequences challenging (Thakore et al., 2015). Second, although CRISPR-based screens best recapitulate physiological conditions when conducted in primary cells, most studies rely on immortalised cell lines that can be engineered to stably express the Cas9 or dCas9 fusion proteins. Indeed, the difficulty of delivering Cas9 has previously hindered efforts to adapt CRISPR screens for primary cells, as the large transgene results in low transduction efficiency when delivered using lentivirus and high toxicity when delivered as a plasmid (Lesueur et al., 2016; Shifrut et al., 2018). CRISPR knockout screens, which only require transient expression of Cas9, have circumvented this issue by delivering Cas9 protein following lentiviral transduction of the sgRNA library (Shifrut et al., 2018; Ting et al., 2018). However, CRISPRi and CRISPRa screens, which induce reversible epigenetic modifications, require more sustained expression of the effector molecules. A recent study has reported a possible solution to this: the authors used an optimised transduction method to efficiently deliver dCas9 fusion proteins via lentiviral vectors into primary human T cells, thereby enabling CRISPRi and CRISPRa screens in these cells (Schmidt et al., 2022). This approach facilitated the identification of genes that could modulate cytokine production in T cells by either activating or interfering with promoter activity – highlighting the potential for similar approaches in other primary cell types to provide insights into key regulatory circuits.

The development of methods to link enhancers to their target genes represents a key advance in efforts to understand how non-coding loci mediate disease risk. Indeed, the ability to identify causal variants within regulatory elements and delineate their downstream functional consequences will build a better understanding of disease mechanisms.

## Translating non-coding SNPs to immune disease mechanisms

Advances in genetic and genomic techniques now provide an unprecedented opportunity to functionally dissect and systematically characterise non-coding genetic variation in immune-mediated disease. Using these approaches, several recent studies have uncovered mechanisms by which causal variants within enhancers perturb regulatory activity and drive immune disease processes. These studies offer important insights into key pathways in immune-mediated disease – some of which may be potential therapeutic targets – and illustrate general principles by which common genetic variants in enhancers can contribute to pathology.

### *IL2RA*: temporal expression alters immune cell differentiation

The interleukin 2 receptor alpha (*IL2RA*) gene has been identified in multiple GWAS studies as a key susceptibility locus for over a dozen immune-mediated diseases (Carr et al., 2009; Ellinghaus et al., 2016; Lowe et al., 2007; Stahl et al., 2010). This locus encodes IL-2Ra, a subunit of the heterotrimeric high-affinity IL-2 receptor that is constitutively expressed by regulatory T (Treg) cells (Box 1) and upon activation by conventional T cells (Sakaguchi et al., 1995; Waysbort et al., 2013). IL-2 signalling plays a critical role in the maintenance of Treg cells, as well as in the expansion and differentiation of naïve T cells (Fontenot et al., 2005; Liao et al., 2011; Liu et al., 2015; Pipkin et al., 2010). Although coding mutations that ablate *IL2RA* expression result in severe immunodeficiency and autoimmunity, the functional consequences of non-coding variation at the *IL2RA* locus have proven more difficult to characterise (Caudy et al., 2007; Goudy et al., 2013; Sharfe et al., 1997). Statistical fine-mapping indicates that the human *IL2RA* locus contains multiple independent signals, including one association refined to a single putative causal variant (rs61839660 C>T) that paradoxically confers risk for Crohn's disease but protection against type 1 diabetes mellitus (Burren et al., 2017; Huang et al., 2017; Maier et al., 2009; Onengut-Gumuscu et al., 2015). This complex pattern of association reflects the complicated regulatory landscape of the locus, which contains a cluster of enhancers that cooperatively tune *IL2RA* expression in response to T-cell receptor (TCR) stimulation and signalling from multiple cytokines, including IL-2 (Busse et al., 2010; John et al., 1996; Li et al., 2017; Liao et al., 2011).

Simeonov et al. (2017) devised a CRISPRa screen to better understand the regulatory landscape of this locus by identifying enhancers that could induce *IL2RA* expression in resting T cells, which was the first time that this methodology had been used to characterise enhancers rather than promoters. By tiling (Box 1) a 178-kilobase region around *IL2RA* in Jurkat-dCas9-VP64 cells (Box 1), the screen uncovered six putative enhancers, including an intronic region containing rs61839660 – the SNP associated with Crohn's disease and type 1 diabetes mellitus. Further investigation in murine CD4^+^ T cells demonstrated that this conserved region is an enhancer that specifically regulates TCR stimulation-induced *IL2RA* expression, and thereby influences the balance of differentiation to either a pro-inflammatory T-helper 17 (Th17) phenotype or a tolerogenic Treg phenotype under IL-2-restricted polarisation conditions. This study additionally showed that, in mice, introduction of the risk allele for rs61839660 leads to a temporal delay in IL-2Ra expression by CD4^+^ T cells. Together, these findings suggest that the causal variant alters Th17/Treg balance by disrupting the timing of *IL2RA* induction – a plausible mechanism by which the variant could contribute to disease. However, the molecular mechanism by which the causal variant delays expression and the reason this has opposing effects on susceptibility to two immune-mediated diseases were not explored in this study. Subsequent work described in a preprint (Simeonov et al., 2020), however, indicated that this intronic enhancer specifically governs IL-2Ra expression in stimulated conventional T cells, whereas a different upstream enhancer controls its expression in Tregs. These observations help parse the enhancer code that governs *IL2RA* expression and provide insights into the nuanced role of IL-2 signalling in maintaining immune homeostasis. The findings can help inform clinical studies by indicating that low-dose IL-2 might induce Treg expansion and suppress aberrant inflammation (Dong et al., 2021).

### *TNFAIP3*: regulation of T-cell activation depends on super-enhancer formation

Among the most challenging disease-associated variants to characterise are those located within loci that contain no protein-coding genes (‘gene deserts’), because there are no immediate indications as to which genes/pathways underpin the associations. Bourges et al. (2020) designed an MPRA to assess regulatory activity of candidate variants from 14 gene deserts associated with ten immune-mediated diseases and leveraged these MPRA findings to investigate disease biology. This study – the first to develop an MPRA for primary immune cells – resolved putative causal variants within these gene deserts via their transcriptional effects in primary human CD4^+^ T cells. The authors investigated the 6q23 gene desert further because of its association with at least six immune-mediated diseases, highlighting the biological importance of this locus, observing that the variant with the greatest expression-modulating effect (rs6927172 C>G) disrupted binding of nuclear factor kappa B (NF-κB; Box 1) at the 6q23 locus in stimulated T cells. The disruption of NF-κB binding reduced enhancer strength across the region. This led to the discovery that the 6q23 region harbours a super-enhancer, which is formed upon T-cell activation and drives expression of tumour necrosis factor alpha-induced protein 3 (*TNFAIP3*). *TNFAIP3* encodes A20, a key inhibitor of NF-κB that suppresses inflammatory responses. This suggested that, in response to cellular stimulation, a regulatory mechanism is induced that limits T-cell activation and prevents uncontrolled inflammation. This study validated the molecular mechanism by which the causal variant at the 6q23 locus disrupts super-enhancer activity using CRISPR in primary CD4^+^ T cells. The authors also demonstrated that deletion of this critical NF-κB binding site leads to unrestrained T-cell responses and thereby provided an explanation for the pleiotropic immune disease risk. These results mirror those of a simultaneous study, which showed that the same rs6927172 risk allele is associated with altered chromatin remodelling and with reduced downstream expression of *TNFAIP3* in stimulated primary human CD4^+^ T cells (Calderon et al., 2019).

The finding that the causal variant at the 6q23 locus broadly compromises the activity of surrounding enhancers (aside from its effect on its cognate enhancer) provides further evidence of the sensitivity of super-enhancers to perturbation of their constituent parts. Although the precise nature and unique functions of super-enhancers remain debated, these elements are generally defined as long stretches of highly active enhancers that are marked by extensive histone modifications and by high densities of Mediator (Box 1), master transcription factors, and other co-factors that regulate the expression of genes central to cell identity (Farooq et al., 2021; Moorthy et al., 2017; Parker et al., 2013; Whyte et al., 2013). Studies that have deleted or mutated component enhancers within super-enhancer loci have found that the effects of such disruption range from mild decreases to near-complete ablation of target gene expression (Hay et al., 2016; Moorthy et al., 2017; Shin et al., 2016). Yet, how disease-associated variants that are enriched in super-enhancers of relevant cell types might affect super-enhancer activity remained largely uncharacterised (Hnisz et al., 2013; Oldridge et al., 2015; Vahedi et al., 2015). Bourges et al. (2020) therefore elucidated a mechanism by which a single variant can disrupt super-enhancer function and contribute directly to multiple diseases.

### *LRRC32*: an enhancer mediates the immunosuppressive activity of Treg cells

Although the 11q13.5 locus is associated with allergic disease, inflammatory bowel disease and type 1 diabetes mellitus, its target gene and the mechanism by which it contributes to disease had not been definitively established (Anderson et al., 2011; Barrett et al., 2008; Esparza-Gordillo et al., 2009; Marenholz et al., 2015; Onengut-Gumuscu et al., 2015). Recently, Nasrallah et al. (2020) identified chromatin features at this locus that were suggestive of enhancer activity in Treg cells. Investigation of this conserved region in mice found that deleting the locus increased susceptibility to colitis and decreased expression of leucine rich repeat containing 32 (*Lrrc32*), the gene encoding glycoprotein A repetitions predominant (GARP). Further study established that the disease-associated locus is a Treg-specific enhancer, which, in response to IL-2 and TCR signalling, is bound by signal transducer and activator of transcription 5 (STAT5) and NF-κB and drives expression of GARP. A small CRISPRa screen in primary human CD4 T cells further indicated that rs11236797, or a nearby variant, is likely to be causal. Colocalisation analysis at this locus also showed that the disease risk alleles colocalise with variants associated with reduced levels of the histone modification H3K27ac in Treg cells. However, the precise mechanism responsible for impaired enhancer activity remains undefined. With these results, the authors nominated GARP as a potential therapeutic target to enhance the immunosuppressive effects of Treg cells. Indeed, a growing number of studies support a key role for GARP in immune regulation, with knockdown or inhibition of GARP reducing Treg-mediated suppression of T-effector cell activity and rare coding mutations in *LRRC32* being associated with primary immunodeficiency and atopic dermatitis (Cuende et al., 2015; Lehmkuhl et al., 2021; Manz et al., 2016; Wang et al., 2010). By characterising the contribution of the 11q13.5 locus to *LRRC32* regulation in Tregs, the study by Nasrallah et al. (2020) helped delineate the pathways that establish Treg-specific expression of *LRRC32*, which had previously been proposed to be caused by either TCR stimulation or signalling from multiple cytokines (Kuhn et al., 2017; Manz et al., 2016; Zhou et al., 2013). This study therefore provides important insights into the regulation of GARP and its central role in Treg cell function.

The examples above illustrate how resolving the biological mechanisms by which non-coding genetic variants contribute to disease can provide important insights into transcriptional regulation, gene function and fundamental immunology, as well as disease mechanisms. Ongoing work to resolve disease associations at non-coding loci promises to yield further insights into immune biology and should uncover novel therapeutic targets.

## Future perspectives

Translating GWAS associations into a better understanding of disease biology remains arguably the most important challenge in modern genetics. Tremendous progress has been made by using recently developed tools and approaches to characterise the functions of the non-coding genome. However, much more work is required to fulfil the potential of GWAS and elucidate the molecular and cellular mechanisms that cause immune-mediated diseases. Several key challenges remain.

First, there is a clear need to develop more accurate cellular models to study disease processes. One area of focus is to adapt high-throughput screening assays and related methods for use in primary human immune cells because these cells best recapitulate effector pathways in disease. Although challenges in obtaining sufficient numbers of primary cells and difficulties in transfecting or transducing these cells have long prohibited their use in high-throughput screens and assays, recent studies have adapted these methods for use in primary human T cells (Bourges et al., 2020; Hiatt et al., 2022; Schmidt et al., 2022). The next step will be to extend these methods into other primary immune cell types that play key roles in disease.

A related area of focus is to identify stimulation conditions that are most relevant to immune-mediated diseases. Appropriate stimulation conditions are essential for studying disease processes because the stimuli used, their dose and duration all determine gene expression programs and cell activation state (Xue et al., 2014). Currently, *in vitro* studies with primary cells often rely on simplistic differentiation and activation conditions – in large part because disease-relevant stimuli have not been fully characterised. Resolving this challenge will therefore ensure that future studies can investigate disease mechanisms using conditions that recapitulate those encountered by cells in diseased tissue.

Second, a better understanding of the molecular mechanisms by which non-coding variants contribute to disease is urgently required. Non-coding variants within enhancers have been shown to predispose to disease by altering transcription factor binding and reducing or augmenting expression of target genes (Bourges et al., 2020; Musunuru et al., 2010). However, we currently have a limited ability to predict the effects of genetic variation on transcription factor binding because many binding motifs are unknown and even well-characterised transcription factors often bind at sequences other than their canonical motifs (Jolma et al., 2013; Weirauch et al., 2014). Moreover, the mechanisms by which altered transcription factor binding leads to differential gene expression are incompletely understood. Indeed, transcription factor binding facilitates processes ranging from formation of enhancer-promoter contacts to chromatin remodelling and DNA methylation, and perturbation of any of these processes can modulate levels of transcription (Di Giammartino et al., 2019; Martin-Trujillo et al., 2020). A further consideration is that some disease-associated non-coding variants lie outside of enhancer regions and must therefore contribute to immunopathology through other mechanisms. The observation that these variants are enriched at sites of post-translational modifications has given rise to several hypotheses of their function. These include the following: (1) that non-coding variants influence RNA splicing (Box 1); (2) that non-coding variants affect RNA editing (Box 1); and (3) that non-coding variants alter the 3′ UTR, which can influence transcript stability and translation (Griesemer et al., 2021; Li et al., 2022, 2016). However, the exact contribution of each of these processes to immune-mediate disease susceptibility remains to be characterised.

Ultimately, addressing these challenges will help translate genetic associations into a deeper knowledge of disease biology. This should reveal novel druggable targets and enable subsequent drug discovery efforts to be directly focused on validated disease mechanisms, thereby improving the treatment of patients with immune-mediated diseases.
